# Investigation of Serum Endocan Levels and Age in Critical Inflammatory Conditions

**DOI:** 10.3390/ijms241210135

**Published:** 2023-06-14

**Authors:** Chrysi Keskinidou, Alice G. Vassiliou, Edison Jahaj, Zafeiria Mastora, Nikolaos Athanasiou, Anastasia Roumpaki, Stamatios Tsipilis, Ioanna Dimopoulou, Stylianos E. Orfanos, Anastasia Kotanidou

**Affiliations:** First Department of Critical Care Medicine & Pulmonary Services, Medical School, National and Kapodistrian University of Athens, Evangelismos Hospital, 10676 Athens, Greece; chrysakes29@gmail.com (C.K.); edisonjahaj@gmail.com (E.J.); zafimast@yahoo.gr (Z.M.); nikolaosathanasiou14@gmail.com (N.A.); ana_roumpaki@yahoo.com (A.R.); stamostsipil@gmail.com (S.T.); idimo@otenet.gr (I.D.); sorfanos@med.uoa.gr (S.E.O.)

**Keywords:** endocan, COVID-19, sepsis, endothelium

## Abstract

Aging negatively affects the endothelium. Endocan (ESM-1), an endothelium-derived soluble proteoglycan, participates in fundamental biological processes of endothelial cells. We aimed to examine the role of endothelial dysfunction and age in poor outcomes in critical illness. ESM-1 levels were measured in the sera of mechanically ventilated critically ill patients, including COVID-19, non-septic, and septic patients. The 3 patient cohorts were divided based on age (≥65 and <65). Critically ill COVID-19 patients had statistically higher ESM-1 levels compared to critically ill septic and non-septic patients. Only in critically ill septic patients were ESM-1 levels higher in older compared to younger patients. Finally, the age-subgrouped patients were further subdivided based on intensive care unit (ICU) outcome. ESM-1 levels were similar in COVID-19 survivors and non-survivors, irrespective of age. Interestingly, only for the younger critically ill septic patients, non-survivors had higher ESM-1 levels compared to survivors. In the non-septic survivors and non-survivors, ESM-1 levels remained unaltered in the younger patients and tended to be higher in the elderly. Even though endocan has been recognized as an important prognostic biomarker in critically ill patients with sepsis, in our patient cohort, increased age, as well as the extent of endothelial dysfunction, seemed to affect its prognostic ability.

## 1. Introduction

Advanced age is a known risk factor for chronic aging-associated lung disease development, as aging negatively affects the proliferative and regenerative capacity of the endothelium, endothelial gene expression, and monolayer integrity [1,2,3]. With increased age, lung functions change, while the immune system also deteriorates, in a process called immunosenescence [4,5]. Endothelial dysfunction is characterized by functional and structural alterations, while the progression of vascular endothelial dysfunction, due to increased life duration, predisposes patients to cardiovascular events [6]. The endothelium plays an important role in the resolution of acute inflammation by regulating leukocyte migration and blood-tissue barrier permeability [7]. When endothelial dysfunction is initiated, a pro-inflammatory phenotype is established. Activated endothelial cells exhibit increased expression and secretion of adhesion molecules and inflammatory mediators, crucial for inflammatory cell recruitment [8].

The endothelial cell specific molecule-1, also known as endocan (ESM-1), is an endothelium-derived soluble proteoglycan that is long considered an important prognostic and severity-assessing biomarker in sepsis, as well as pulmonary and vascular diseases [9,10,11]. ESM-1 participates in fundamental biological processes of endothelial cells, including cell adhesion, migration, proliferation, and neoangiogenesis. Following the activation of endothelial cells by viral, bacterial, and inflammatory stimuli, ESM-1 synthesis is remarkably increased. The role of ESM-1 during the acute phase of inflammation is in the binding and rolling of leukocytes to the site of inflammation [12].

In a previous study we showed that ESM-1 levels were much higher on intensive care unit (ICU) admission in critically ill COVID-19 non-survivors compared to survivors, who had not received dexamethasone. In the dexamethasone-treated patients, ESM-1 levels were reduced, and could no longer differentiate survivors from non-survivors [13]. The aim of this study was to explore whether ESM-1 levels can act as a prognostic factor of poor outcomes, in both older and younger patients, in different critically ill patient groups.

## 2. Results

### 2.1. Clinical Characteristics of the Study Population

The demographic and clinical characteristics of our patients are summarized in Table 1. All patients were critically ill, mechanically ventilated, with or without COVID-19. Critically ill COVID-19 patients were older than critically ill septic and non-septic patients, and the majority (78.6%) had comorbidities, mostly arterial hypertension and diabetes. Moreover, critically ill COVID-19 patients had a higher mortality rate (46.7%) compared to both critically ill septic and non-septic patients. Most critically ill septic and non-septic patients were admitted to the ICU mainly due to trauma (43% and 53.5%, respectively). The remaining diagnoses were surgery (34% and 22.5%, respectively, mostly emergency), and medical diagnoses (23% and 24%, respectively, mainly central nervous system pathologies). The critically ill non-septic patients had the lowest mortality rates (9.7%). The Berlin definition criteria for acute respiratory distress syndrome (ARDS) diagnosis were met in 84.4% of the critically ill COVID-19 patients, 58.2% of the critically ill septic patients, and 43.9% of the critically ill non-septic patients. In the critically ill septic patients, the median day of sepsis development during their ICU stay was 7 [interquartile range (IQR): 4–11], of whom 25 also developed septic shock on day 14 (IQR: 8–23). Of note, all critically ill septic patients had a respiratory infection caused by Gram-negative bacteria (63% *Acinetobacter baumanii*, 16.5% *Klebsiella pneumoniae*, 7.6% *Acinetobacter baumanii* and *Klebsiella pneumoniae*, 5.2% *Serratia marcescens*, 5.2% *Enterobacter* species, and 2.5% *Pseudomonas aureginosa*).

ESM-1 levels were measured in all patients recruited in the study, within the first 24 h of ICU admission (critically ill COVID-19 patients and critically ill non-septic patients), or 6 h following sepsis diagnosis (critically ill septic patients). Within the critically ill COVID-19 group, ESM-1 levels did not differ between dexamethasone-treated and non-treated patients [1477 (939–2373) pg/mL vs. 1885 (1079–3127) pg/mL, *p* = 0.11, respectively]; since there was no statistically significant difference, we pooled the patients into the same group (critically ill COVID-19 patients). Critically ill COVID-19 patients had statistically higher ESM-1 levels compared to critically ill septic and non-septic patients [1527 (951–2406) pg/mL vs. 652 (384–937) pg/mL and 753 (370–1232) pg/mL, *p* < 0.0001, respectively]. ESM-1 levels in the critically ill septic patients were comparable to those measured in the non-septic patients and did not exhibit a statistically significant difference. ESM-1 levels positively correlated with hypertension in the critically ill COVID-19 patients (r_s_ = 0.2231, *p* = 0.0054), while in the critically ill septic patients ESM-1 levels strongly correlated with age (r_s_ = 0.3961, *p* = 0.0003) and the SOFA score (r_s_ = 0.2291, *p* = 0.04).

### 2.2. ESM-1 Levels in Critically Ill COVID-19, Septic, and Non-Septic Patients Based on Age

**Figure 1:**
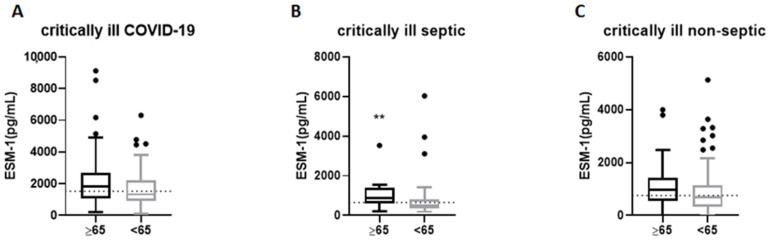
ESM-1 levels in critically ill COVID-19, septic and non-septic patients based on age. ESM-1 levels were measured in (**A**) 154 critically ill COVID-19 (≥65, N = 89 and <65, N = 65), (**B**) 79 critically ill septic (≥65, N = 24 and <65, N = 55) and (**C**) 164 critically ill non-septic patients (≥65, N = 45 and <65, N = 119) upon ICU admission, in the first 24 h for the critically ill COVID-19 and non-septic patients, and in the first 6 h of sepsis diagnosis for septic patients. Patients were divided into two subgroups based on their age: patients ≥65 years old and <65 years old. The non-parametric Mann–Whitney test was performed for two group comparisons. Data are presented as box plots. Line in the box, median value; box edges, 25th to 75th centiles; whiskers, range of values; bullets, outliers. In each graph, the dashed horizontal line indicates the median value of each patient group. ** *p* < 0.01. ESM-1 = Endocan.

The three patient groups were divided into two subgroups based on their age: patients ≥ 65 years old and <65 years old [14]. In COVID-19, older patients (≥65, N = 89) tended to have higher ESM-1 levels compared to younger patients (N = 65), albeit not statistically significant [1825 (1056–2676) pg/mL vs. 1328 (906–2207) pg/mL, respectively, *p* = 0.051; Figure 1A]. To clarify whether dexamethasone treatment has an age-dependent effect on ESM-1 levels in COVID-19 patients, we divided the patients into dexamethasone-treated and non-treated groups. We found that the non-treated patients ≥ 65 had higher ESM-1 levels [2253 (1840–4352) pg/mL] compared to the younger [1320 (906–2126) pg/mL; *p* = 0.006), while in the dexamethasone-treated group, ESM-1 levels did not differ between older [1547 (943–2518) pg/mL] and younger patients [1340 (869–2245) pg/mL; *p* = 0.28]. Hence, it seems that dexamethasone treatment has an age-dependent effect on ESM-1 levels, since only in the older patients were ESM-1 levels reduced.

Elderly critically ill septic patients (≥65, N = 24) exhibited higher ESM-1 levels compared to younger septic patients (N = 55) [861 (623–1397) pg/mL vs. 520 (352–821) pg/mL, respectively, *p* = 0.0013; Figure 1B]. Finally, critically ill non-septic patients had comparable ESM-1 levels in the two age subgroups [(≥65, N = 45), 978 (554–1416) pg/mL vs. (<65, N = 119), 696 (342–1154) pg/mL, *p* = 0.08; Figure 1C].

### 2.3. ESM-1 Levels in Survivors and Non-Survivors Based on Age

**Figure 2:**
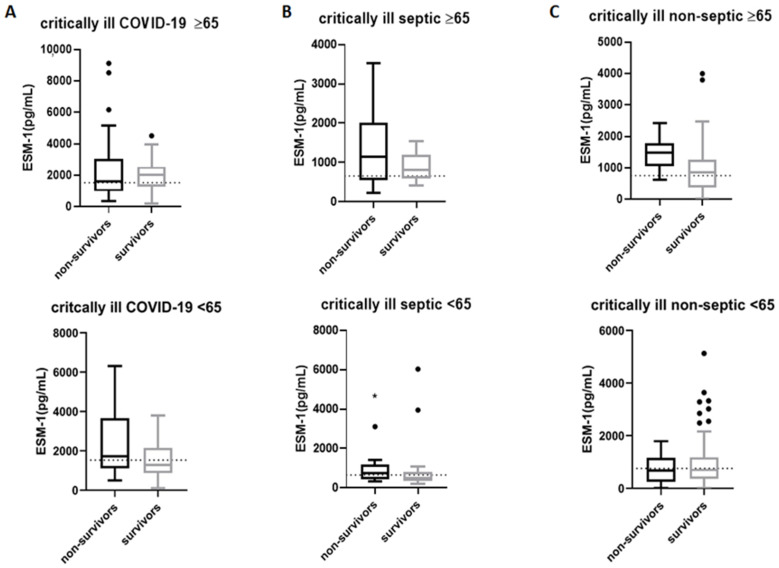
ESM-1 levels in survivors and non-survivors of the age subgroups. The three critically ill patient groups, (**A**) COVID-19, (**B**) septic, (**C**) non-septic, were divided into two subgroups based on age: patients ≥ 65 years old and <65 years old, and were further subdivided based on their ICU outcome into survivors and non-survivors. The non-parametric Mann–Whitney test was performed for two group comparisons. Data are presented as box plots. Line in the box, median value; box edges, 25th to 75th centiles; whiskers, range of values; bullets, outliers. In each graph, the dashed horizontal line indicates the median value of each patient group. * *p* < 0.05. ESM-1 = Endocan.

Subsequently, we aimed to examine whether ESM-1 levels differed between survivors and non-survivors of the age subgroups mentioned above. To this end, we further subdivided our age subgroups into survivors and non-survivors and examined ESM-1 levels between the former and the latter. As shown in Figure 2A, ESM-1 levels in COVID-19 patients were similar in survivors and non-survivors, irrespective of age [≥65; 2039 (1274–2549) pg/mL (N = 35) vs. 1612 (990–3053) pg/mL (N = 54), respectively, *p* = 0.82; Figure 2A], and [<65; 1285 (867–2151) pg/mL (N = 47) vs. 1726 (1109–3663) pg/mL (N = 18), respectively, *p* = 0.093; Figure 2A]. Interestingly, in the ≥65-years-old critically ill septic patients, survivors and non-survivors had similar serum levels of ESM-1 [805 (594–1097) pg/mL (N = 18) vs. 1148 (554–2018) pg/mL (N = 6), respectively, *p* = 0.494; Figure 2B], yet in younger critically ill septic patients (<65), non-survivors had higher ESM-1 levels compared to survivors [760 (429–1176) pg/mL (N = 10) vs. 511 (332–789) pg/mL (N = 45), respectively, *p* = 0.045; Figure 2B]. In the non-septic survivors and non-survivors, ESM-1 levels remained unaltered in the younger critically ill patients [696 (364–1176) pg/mL (N = 109) vs. 679 (247–1157) pg/mL (N = 10), respectively, *p* = 0.59; Figure 2C], and tended to be higher in the elderly non-survivors [867 (376–1267) pg/mL (N = 38) vs. 1494 (1047–1786) pg/mL (N = 7), *p* = 0.056; Figure 2C].

## 3. Discussion

As populations are becoming older in all regions of the world, the age of patients admitted to the ICU is also increasing. Expanding our understanding on how age-related changes affect disease risk and severity could provide important information for improving existing therapies and developing new therapeutic strategies. In the present study, we measured the serum levels of ESM-1 in three different groups of critically ill patients. The patient groups were divided based on their age. In COVID-19, older patients (≥65) tended to have higher ESM-1 levels compared to younger patients. Older critically ill septic patients exhibited higher ESM-1 levels compared to younger septic patients, whereas critically ill non-septic patients had comparable ESM-1 levels in the two age subgroups. When patients were subcategorized based on their age and their outcome, only younger septic non-survivor patients had statistically significant higher levels of ESM-1, while elder critically ill non-septic patients tended to have higher levels.

Endothelial dysfunction, or endotheliopathy, is characterized by a shift of the physiological endothelial regulation to a pro-inflammatory and pro-thrombotic phenotype. Endothelial disruption and damage are important pathophysiologic mechanisms in critical illness and the degree of vascular endothelial cell injury is associated with disease severity and poor outcome. The role of endotheliopathy has been thoroughly examined in acute critical illness, such as trauma, sepsis, and ARDS [15,16]. Moreover, endothelial activation and dysfunction are considered major components of COVID-19 pathogenesis, and COVID-19-associated endotheliopathy has been associated with disease severity and worse clinical progression [17,18,19,20]. The observed elevated ESM-1 levels in the present study might indicate the extended disruption of vascular integrity following the severe acute respiratory syndrome coronavirus 2 (SARS-CoV-2) infection. Endothelial markers of inflammation, endothelial cell activation and injury, coagulation, and fibrinolysis have been assessed in patients with severe COVID-19, evaluating their prognostic value in predicting disease severity and negative outcomes [13,21,22].

Many studies have assessed the value of ESM-1 as a diagnostic and prognostic marker in COVID-19. ESM-1 has been recognized as a novel biomarker capable of predicting COVID-19 severity, outcome, and related complications [21,22,23,24]. In a previously published study, we demonstrated that on ICU admission, ESM-1 levels were significantly higher in non-survivors compared to survivors in COVID-19 patients not treated with dexamethasone. Even though ESM-1 levels in critically ill COVID-19 patients treated with dexamethasone were comparable with non-treated patients, in the dexamethasone-treated group, ESM-1 could no longer distinguish survivors from non-survivors [13]. In the present study, our COVID-19 patients consisted of both dexamethasone-treated and non-treated patients, and therefore, likewise, ESM-1 could not differentiate survivors from patients with poor outcomes. Innate immune defenses against pathogens also decline with age. Elderly patients are more susceptible to infection by SARS-CoV-2, and, therefore, are considered a high-risk group [25]. In our critically ill COVID-19 cohort, there was no statistically significant difference in ESM-1 levels between survivors and non-survivors in both age subgroups. We believe that dexamethasone treatment is partially responsible for this. However, dexamethasone treatment did not reduce ESM-1 levels in a statistically significant manner within the critically ill COVID-19 group. It is plausible that, even after dexamethasone treatment, the extent of endotheliopathy due to SARS-CoV-2 infection is very persistent in COVID-19 patients, regardless of their outcome and their age, and therefore the prognostic value of ESM-1 in our critically ill COVID-19 cohort was diminished.

A great number of studies have reported that ESM-1 is a novel biomarker for sepsis severity stratification and mortality [26,27,28,29]. Furthermore, an increase in ESM-1 levels has been associated with worsening of the patients’ condition [30]. It is worth noticing that, in many of these studies evaluating ESM-1 levels in sepsis, the septic cohort usually consists of patients younger than 65 years old. Herein, we also observed that younger critically ill septic non-survivors had statistically significant higher ESM-1 levels compared to survivors. However, with regards to older critically ill septic patients, there was no statistically significant difference observed between survivors and non-survivors. In our critically ill septic patients it seems that age might affect the prognostic ability of ESM-1. Elderly patients are at greater risk for cardiovascular diseases and have deteriorated immune responses, and therefore are at greater risk of developing sepsis or other complications during their ICU stay.

In our older critically ill non-septic group, non-survivors had a tendency for higher ESM-1 levels compared to survivors. There was no difference between survivors and non-survivors among the younger critically ill non-septic patients. Our results might suggest that, contrary to our critically ill septic patients, in our younger critically ill non-septic patients, ESM-1 may not be used as a prognostic biomarker for mortality. Elderly patients should be included in studies examining novel prognostic sepsis biomarkers so that the biomarker’s prognostic value is evaluated in a more realistic clinical setting and age-inclusive manner.

It is important to mention the limitations of our study. This is a single-center study, with a moderate number of patients, which might weaken statistical conclusion validity. Moreover, the COVID-19 patients were older. Furthermore, in the current study we did not examine any mechanisms underlying endothelial injury and vascular dysfunction. A larger number of patients, including elderly patients, in a cross-center study could provide us with a more accurate picture of the relationship between endothelial dysfunction and age. However, it is important to highlight the strengths of the present study. First of all, we examined three different groups of critically ill patients: COVID-19, septic and non-septic patients. To the best of our knowledge, this is the first study comparing the levels of ESM-1, an endothelial biomarker of glycocalyx shedding, in three different critically ill groups. Secondly, we evaluated the prognostic value of ESM-1 in elderly patients, while many published studies include <65 years old critically ill patients [31,32].

## 4. Materials and Methods

The study was approved by the Evangelismos Hospital Research Ethics Committee (129/19-3-2020). Prior to study enrollment, all patients’ next-of-kin provided informed written consent, and all procedures were in accordance with the Helsinki Declaration. Demographic data, patients’ symptoms and vital signs, comorbidities, and laboratory findings were recorded for each patient recruited in the study. Moreover, ICU scoring systems, including acute physiology and chronic health evaluation (APACHE II) and sequential organ failure assessment (SOFA) scores, were calculated upon admission. The Berlin definition was used to assess ARDS [33] and the Sepsis-3 definitions for septic patients’ characterization [34].

The study included 154 consecutive critically ill, mechanically ventilated patients with confirmed SARS-CoV-2 infection (*critically ill COVID-19 patients*), diagnosed by real-time reverse transcription PCR (RT-PCR) in nasopharyngeal swabs, and 244 SARS-CoV-2-negative critically ill, mechanically ventilated patients. Of these, 79 fulfilled the criteria for sepsis diagnosis during their ICU stay (*critically ill septic patients*), while 164 did not develop sepsis (*critically ill non-septic patients*). Of the 154 critically ill COVID-19 patients, 37 (24%) had not received dexamethasone treatment, while the remaining 117 (76%) had received one dose of dexamethasone (6 mg). Overall ICU mortality was defined as the study outcome. In order to examine whether older age affects ESM-1 levels, patients were grouped based on age (≥65 and <65 years) [14].

Three milliliters (3 mL) of venous blood was collected and prepared for serum collection within the first 24 h of ICU admission (critically ill COVID-19 patients and critically ill non-septic patients), or 6 h following sepsis diagnosis (critically ill septic patients).

Soluble levels of ESM-1 were measured in serum samples using the enzyme-linked immunosorbent assay (ELISA) kit by Origene (OriGene Technologies, Inc., Rockville, MD, USA) according to the manufacturers’ instructions. All assays were performed by the same researcher who was blinded to the samples measured.

Statistical analysis was performed using GraphPad Prism, v8.0 (GraphPad Software, San Diego, CA, USA). Based on the data distribution, normally distributed variables are presented using the mean ± SD and variables with skewed distributions are presented using median (IQR). The chi-square statistical test was used to compare qualitative variables, while two group comparisons for skewed data were performed by the non-parametric Mann–Whitney test. Three group comparisons were performed by the Kruskal–Wallis test followed by Dunn’s multiple comparisons test. The Spearman’s correlation coefficient was used to perform correlations. Following two-sided testing, *p*-values were calculated; *p*-values < 0.05 were considered to be significant.

## 5. Conclusions

In the present study, we observed that the prognostic value of ESM-1 in our critically ill patients is impeded by older age and the extent of endothelial dysfunction. ESM-1 does remain an important prognostic biomarker in sepsis. However, when assessing the prognostic value of a novel biomarker, it is critical to consider age groups and disease severity levels that reflect real-life clinical settings. Unfortunately, none of the existing biomarkers can serve alone as a prognostic tool for every disease and severity. ESM-1 is a very important biomarker in sepsis and a novel biomarker in COVID-19; however, in our study it seems that its prognostic value was restricted by older age and the severity of the symptom manifestations. It is important to point out, however, that this finding is related to our cohort; our study design and the data collected do not allow us to generalize our results. Further studies with more subjects are needed to validate our finding.

## Figures and Tables

**Table 1:** Demographics and clinical characteristics of the study population.

Characteristics	Critically Ill COVID-19 Patients	Critically Ill Septic Patients	Critically Ill Non-Septic Patients
Number of patients, N	154	79	164
Age (years), (mean ± SD)	66 ± 13.0	59 ± 13.0 ***	53 ± 18.0 ****
Sex, N (%) Male Female	116 (75.0) 38 (25.0)	56 (70.9) 23 (29.1)	118 (71.9) 46 (28.1)
Clinical characteristics			
Diagnosis, N (%)			
Medical	154 (100.0)	18 (22.8) ****	39 (23.8) ****
Surgical	0 (0.0)	27 (34.2)	37 (22.6)
Elective	0	6	8
Emergency	0	21	29
Trauma	0 (0.0)	34 (43.0)	88 (53.6)
Comorbidities, N (%)	121 (78.6)	35 (44.3) ****	59 (35.9) ****
Hypertension	73	24	34
Diabetes	31	6	10
APACHE II, (median, IQR)	15 (13–18)	17 (13–21) *	14 (11–18)
SOFA, (median, IQR)	5 (4–6)	6 (5–9) **	6 (5–8) **
PaO_2_/FiO_2_ (mmHg), (median, IQR)	257.1 (198.0–281.0)	246 (175.4–330.0)	321.6 (234.0–406.6) ****
ARDS, N (%)	130 (84.4)	46 (58.2) ****	72 (43.9) ****
CRP (mg/dL), (mean ± SD)	13.3 ± 9.1	12.8 ± 7.4	6.7 ± 6.5 ****
PCT (ng/mL), (median, IQR)	0.30 (0.11–1.30)	0.17 (0.11–0.56)	0.24 (0.10–1.03)
Endothelial Marker			
ESM-1 (pg/mL), (median, IQR)	1527.0 (951.4–2406.0)	651.6 (383.5–936.6) ****	753.4 (369.6–1232.0) ****
Outcomes			
Mortality, N (%)	72 (46.7)	16 (20.3) ****	16 (9.7) ****
LoS in the ICU (days), (median, IQR)	20 (13–37)	30 (23–40) **	15 (7–30) ***

Data are expressed as the number of patients (N) and percentages of total related variable (%). Normally distributed variables are presented as mean ± standard distribution (SD) and skewed data are presented as median (IQR). The study included 3 patient groups, namely critically ill COVID-19 patients, critically ill septic, and non-septic patients. Three group comparisons were performed by Kruskal–Wallis ANOVA, followed by Dunn’s multiple comparisons test. The chi-square test was used to analyze nominal data. Clinical characteristics, vital signs, laboratory data, and ESM-1 levels were measured on ICU admission (within 24 h) for COVID-19 and critically ill non-septic patients, while for critically ill septic patients, measures were performed within 6 h of sepsis diagnosis. Outcomes were recorded at ICU discharge or upon patients’ death. Abbreviations: APACHE = Acute physiology and chronic health evaluation; ARDS = Acute respiratory distress syndrome; CRP = C-reactive protein; ESM-1 = Endocan; LoS = Length of stay, PCT = procalcitonin, and SOFA = Sequential organ failure assessment. *p*-values indicate differences with the critically ill COVID-19 group. * *p*-value < 0.05; ** *p*-value < 0.01; *** *p* < 0.001 and **** *p*< 0.0001 compared to the COVID-19 group.

## Data Availability

Available upon reasonable request.
